# p53, ROS and senescence in the control of aging

**DOI:** 10.18632/aging.100189

**Published:** 2010-08-16

**Authors:** Arnaud Vigneron, Karen H Vousden

**Affiliations:** The Beatson Institute for Cancer Research, Glasgow, G61 1BD, UK

## Abstract

Abstract: In addition to its function as a tumour suppressor, p53 is also involved in an increasing number of pathology associated with aging. Several activities of p53 appear contribute to its role in aging; one function that might be particularly relevant in this context is the regulation of senescence. The control of ROS and senescence by p53 may help to explain how p53 can function to both restrain and promote aging.

p53 functions as a longevity assurance gene (by virtue of its strong tumor suppressor activity) and a regulator of aging. In several mouse models, persistent low-level activation of p53, either through deregulated expression of p53 itself or in response to constitutive stress like DNA damage/telomere erosion, leads to premature aging [1,2]. However, mice with normal basal p53 levels that have been engineered to a show a heightened ability to mount a p53 response show a very strong resistance to tumourigenesis without evidence of premature aging [3]. Indeed, in several of these models a decreased level of aging related damage is observed, indicating that p53 may also help to promote longevity. The control of aging reflects numerous activities of p53, including the modulation of the IGFR pathway through interplay between full-length p53 and N-terminally truncated splice variants of p53 [4] and the ability of p53 to restrict stem cell function [5]. p53 is also a key regulator of senescence, a central stress response that plays an important role in tumour suppression, but may also help to promote cancer development by inducing an inflammatory response [6]. The ability to control senescence is consistent with p53's function in restraining cancer development, but can the mechanisms through which p53 regulates senescence also contribute to the control of aging? Induction of senescence by p53 is associated with the regulation of p53-dependent genes that can participate in cell cycle arrest. While depletion of these components can impact senescence induction - supporting their role in mediating this response - the inhibition of cell cycle progression alone does not explain how this arrest can be turned into the definitive and permanent proliferation block that is characteristic of senescence. Furthermore, despite the clear documentation of p53's ability to induce senescence, more recent evidence shows that p53 can also function to inhibit senescence while promoting cell cycle arrest [7]. So how can p53 both suppress and promote senescence? An important component of this may be the ability of p53 to control cell growth and metabolic stressthrough different pathways, including the regulation of ROS levels and the activity of mTOR (Figure 1). The ability of p53 to promote ROS production has been shown to participate in the induction of apoptosis by p53 [8]. But ROS are also known to be critical for senescence [9] and the p53 target genes that increase ROS may also play an important role in senescence induction. However, p53 also promotes the expression of a number of antioxidant genes, accounting for p53's ability to control oxidative stress in cells and mice [10]. So p53's ability to decrease and increase oxidative stress likely contributes to its dual effect on senescence. Another factor that influences the outcome to p53 activation is mTOR. While mTOR is normally associated with cell growth, activation of mTOR can contribute to and be essential for certain types of senescence [11,12], and the maintenance of mTOR signalling under conditions of cell cycle arrest leads to senescence in cultured cells [13]. p53 inhibits the mTOR pathway at several levels [14], contributing to the anti-senescence activity of p53 [15]. Furthermore, mTOR can be activated by ROS [16], so p53's antioxidant activities may reinforce the dampening of mTOR and senescence (Figure 1).

One of the main responses to mTOR inhibition is the induction of autophagy, a response that allows survival under conditions of nutrient deprivation. There are several possible links between autophagy and senescence. Inhibition of autophagy results in the accumulation of protein aggregates, ER stress and mitochondrial dysfunction, each of which could promote senescence. However, other studies suggest that autophagy may be required for an efficient senescence response [17]. In either case, the ability of p53 to both enhance and inhibit autophagy [18] provides a further mechanism for the modulation of senescence.

The activity of p53 is regulated through many mechanisms, but of particular interest with respect to the control of senescence and aging is a role for the histone deacetylase Sirt1, whose expression is strongly down regulated in senescent cells [19]. In contrast nutrient deprivation, which inhibits mTOR and can impede cellular senescence [13], has been shown to increase Sirt1 levels [20]. One way in which Sirt1 functions is to deacetylate p53, modulating p53 activity and impeding decreasing senesence [21]. Deactylation inhibits p53's ability to transcriptionally activate some, but not all, target genes - including those involved in apoptosis induction, ROS production [22,23], and presumably also senescence (Figure 1). The presence of a chronic DNA damage response (as may be seen in cancer cells), which is linked to the induction of senescence [24], can directly increase p53 acetylation by inducing the phosphorylation of the N-terminus of p53 and so promoting the interaction with the acetyl transferases CBP/p300. Mouse models have shown that expression of phosphorylation resistant p53 inhibits the induction of senescence [25], while cells harbouring p53 with acetyl-mimicking mutations of the last seven lysine residues have an accelerated entry into senescence and are very resistant to senescence bypass [26], although the cell cycle arrest response in these cells remains normal. Phosphorylation and acetylation of p53 is also seen to be important during Ras-induced or replicative senescence [27,28]. Under these circumstances, it would seem that deacetylation of p53 by Sirt1 impedes the induction of senescence, as well as apoptosis. Taken together there is good evidence that acetylation of p53 promotes sense-cence and apoptosis, so inhibitors of the deacetylation enzymes might be useful drugs for the reactivation of these p53 responses for cancer therapy [29].

**Figure 1.**
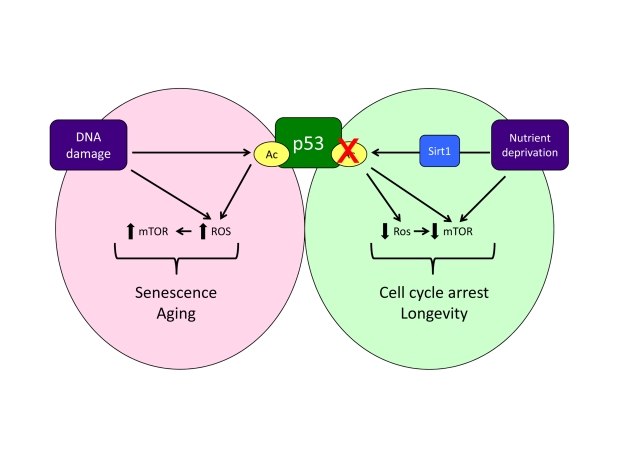
A model of how acetylation, oxidative stress and mTOR activity might influence the response to p53. Note that this model does not account for all published observations (e.g. reduction of the initial burst of mTOR activity during oncogene induced senescence [17]) and represents an oversimplification of these signalling pathways.

Several of the mechanisms implicated in the induction of senescence by p53 have also been linked to the regulation of longevity. Induction of mTOR and oxidative stress - and the complex interplay between them - is associated with aging [16] and Sirt1 is emerging as a key supporter of longevity in many organisms [30]. The induction of cellular senescence itself may result in loss of tissue renewal and architecture, organ dysfunction and organismal aging [31], while autophagy can protect from aging [32]. So it seems reasonable to propose that p53's ability to influence aging is reflected - at least in part - by the mechanisms through which p53 controls senescence.But as we have discussed, p53 can promote and impede both senescence and aging - so which output prevails? The answer is not yet clear, but one determining factor may be the type or extent of the p53-inducing stress. Current models suggest that mild or constitutive stress induced by normal growth and proliferation lead to p53-induced antioxidant and repair functions, while strong or persistent p53 activity may tip the balance towards the induction of apoptosis or senescence, thereby favoring aging. The mouse models also clearly suggest that inappropriate p53 activity promotes aging while a robust but normally regulated p53 response protects from the aging process. One prediction of this model is that the persistent stress encountered in tumors would favor p53-induced senescence over a more transient cell cycle arrest - and indeed the activation of p53 in established tumors has been shown to promote senescence in some tissue types [33].

p53 is emerging as an important, but complex, player in the regulation of senescence and longevity. The ability of p53 to both activate and inhibit senescence is reflected in the ability to promote and inhibit oxidative stress and autophagy, and the ultimate establishment of senescence or quiescence is highly dependent on collaborating factors such as mTOR activity or oxidative stress. Ultimately, these bipolar activities of p53 become manifest in the contradictory effects on longevity and aging. p53 based cancer therapies may be rendered more effective by an increased propensity of transformed cells to undergo senescence, compared to normal cells. However, the idea that p53 can both promote and prevent aging adds even more spice to the consideration of how to use drugs that can induce or inhibit p53 activity.
